# Plasma tumour DNA as an early indicator of treatment response in metastatic castration-resistant prostate cancer

**DOI:** 10.1038/s41416-020-0969-5

**Published:** 2020-07-16

**Authors:** Vincenza Conteduca, Daniel Wetterskog, Emanuela Scarpi, Alessandro Romanel, Giorgia Gurioli, Anuradha Jayaram, Cristian Lolli, Delila Gasi Tandefelt, Giuseppe Schepisi, Chiara Casadei, Anna Wingate, Federica Matteucci, Giovanni Paganelli, Enrique Gonzalez-Billalabeitia, Francesca Demichelis, Ugo De Giorgi, Gerhardt Attard

**Affiliations:** 1 Istituto Scientifico Romagnolo per lo Studio e la Cura dei Tumori (IRST) IRCCS, 47014 Meldola, Italy; 2 University College London Cancer Institute, London, WC1E 6DD UK; 3 Department of Cellular, Computational and Integrative Biology, University of Trento, 38123 Trento, Italy; 4 Department of Urology, Sahlgrenska Academy, University of Gothenburg, Gothenburg, Sweden; 5 Hospital Universitario Morales Meseguer-IMIB and UCAM, Murcia, Spain

**Keywords:** Predictive markers, Prostate cancer

## Abstract

**Background:**

Plasma tumour DNA (ptDNA) levels on treatment are associated with response in a variety of cancers. However, the role of ptDNA in prostate cancer monitoring remains largely unexplored. Here we characterised on-treatment ptDNA dynamics and evaluated its potential for early assessment of therapy efficacy for metastatic castration-resistant prostate cancer (mCRPC).

**Methods:**

Between 2011 and 2016, 114 sequential plasma samples from 43 mCRPC abiraterone-treated patients were collected. Targeted next-generation sequencing was performed to determine ptDNA fraction. ptDNA progressive disease was defined as a rise in the fraction compared to the pre-treatment.

**Results:**

A ptDNA rise in the first on-treatment sample (interquartile range (IQR) 2.6–3.7 months) was significantly associated with increased risk of early radiographic or any prostate-specific antigen (PSA) rise (odds ratio (OR) = 15.8, 95% confidence interval (CI) 3.5–60.2, *p* = 0.0002 and OR = 6.0, 95% CI 1.6–20.0, *p* = 0.01, respectively). We also identified exemplar cases that had a rise in PSA or pseudoprogression secondary to bone flare but no rise in ptDNA. In an exploratory analysis, initial ptDNA change was found to associate with the duration of response to prior androgen deprivation therapy (*p* < 0.0001) but not to prior taxanes (*p* = 0.32).

**Conclusions:**

We found that ptDNA assessment for therapy monitoring in mCRPC is feasible and provides data relevant to the clinical setting. Prospective evaluation of these findings is now merited.

## BACKGROUND

Prostate cancer is one of the most frequently diagnosed cancers in men and the second-leading cause of cancer-related death worldwide.^1^ In recent years, treatment of metastatic castration-resistant prostate cancer (mCRPC) has changed with the introduction of different therapeutic options and optimising clinical benefit has become progressively challenging.^2^ Treating patients with ineffective therapies leads to unnecessary toxicity while also allowing symptomatic cancer progression. However, early discontinuation of a drug that is providing some degree of clinical benefit relative to other available modalities can also be disadvantageous. Currently, standard disease evaluation recommended by the Prostate Cancer Clinical Trials Working Group 3 (PCWG3) guidelines^3^ specifies imaging tests and prostate-specific antigen (PSA) assessments: these guidelines now also highlights the need for the evaluation of progressive disease (PD) at established timepoints using liquid biopsies to better characterise disease biology and identify potential predictive molecular biomarkers.

Circulating, cell-free DNA, often termed plasma tumour DNA (ptDNA) in the oncology setting, is rapidly becoming a quantitative and qualitative analyte to measure treatment efficacy.^4^ Its promise as a minimally invasive biomarker is supported by the high concordance of detected genomic alterations with matched metastatic biopsies.^5,6^

The amount of tumour DNA in the pool of cell-free DNA is called the tumour fraction or tumour content and ranges greatly from almost undetectable up to 90%.^7^ The presence of a low tumour fraction could be a limitation for plasma DNA analyses. However, the introduction of new genomic technologies with high sensitivity and specificity, including next-generation sequencing (NGS),^8^ has greatly contributed to the study of ptDNA.^9,10^

In prostate cancer, we and others have shown an association between pre-treatment ptDNA fraction assessed by NGS and clinical outcome.^7,11–14^ A recent randomised phase 2 study (NCT02125357)^15^ combining whole-exome and/or deep targeted sequencing on ptDNA samples from 202 patients with first-line mCRPC treated with abiraterone or enzalutamide showed that genomic alterations in *BRCA2*, *ATM*, *TP53* and *AR* were associated with treatment outcome and low pre-treatment ptDNA fraction was correlated with a good prognosis. However, the role of ptDNA change in response to treatment and its relationship with PSA and imaging assessment in prostate cancer monitoring is largely unexplored. Here we aimed to determine if plasma DNA dynamics could be used as an early assessment of therapy efficacy for mCRPC.

## METHODS

### Study design and patient cohort

Plasma samples were prospectively collected with the primary objective of biomarker evaluation approved by the Institutional Review Board of Istituto Scientifico Romagnolo per lo Studio e la Cura dei Tumori (IRST) IRCCS, Meldola, Italy (REC 2192/2013). Participants had histologically confirmed prostate adenocarcinoma without neuroendocrine differentiation, PD despite “castration levels” of serum testosterone (<50 ng/dL), ongoing luteinizing hormone-releasing hormone (LHRH)-analogue treatment or prior surgical castration. Patients received treatment with abiraterone 1 g once a day and prednisone 5 mg twice daily as first- or second-line therapy. Abiraterone was administered continuously until evidence of PD or unacceptable toxicity. Serum PSA was evaluated within 3 days of beginning therapy and monthly thereafter. Radiographic disease was assessed with the use of computed tomography and bone scan at the time of screening and every 12 weeks on treatment. The study was conducted in accordance with the Declaration of Helsinki and the Good Clinical Practice guidelines of the International Conference of Harmonisation. Written informed consent was obtained from all patients.

### Circulating tumour DNA fraction analysis

Serial blood samples were collected pre-treatment and, when possible, on treatment and at progression. Circulating DNA was extracted from 1 to 2 mL of plasma from each patient using the QIAamp Circulating Nucleic Acid Kit (Qiagen) and quantified using the high-sensitivity Quant-iT PicoGreen double-stranded DNA Assay Kit (Invitrogen) as previously described.^7,12^

Targeted NGS was performed on the PGM Ion Torrent using a 316 or 318 Chip for a target of 1000× coverage for *AR*, *TP53*, *FOXA1*, *CYP17A1*, *SPOP* and regions of loss in prostate cancer. The ptDNA fraction for each plasma sample was estimated using the CLONET computational tool estimating genomic deletions.^7^ This method to determine ptDNA fraction was recently shown to significantly correlate with an orthogonal method of ptDNA assessment involving targeted methylome NGS.^11^

### Statistical analysis

The primary objective of the study was to compare ptDNA fraction changes with radiographic/biochemical response after 3-month therapy. The secondary objective was to evaluate the role of ptDNA in tumour monitoring from starting abiraterone treatment to PD defined as biochemical and radiographic according to the PCWG3 criteria.^3^ PtDNA progression was considered as any increase of ptDNA from baseline value. Radiographic progression-free survival (PFS) was calculated from the first day of therapy to the date of PD or death, whichever occurs first, or last tumour evaluation. Waterfall plots showed the magnitude of ptDNA fraction or PSA decline in patients with different tumour responses at 3-month therapy (response, stable disease, progression). The odds ratios (ORs) and 95% confidence interval (CI) were calculated using logistic regression analysis. All *p* values were two sided and a *p* < 0.05 was considered as statistically significant. Statistical analyses were performed with the SAS 9.4 software (SAS Institute, Cary, NC, USA).

## RESULTS

### Patient and plasma sample characteristics

**Fig. 1:**
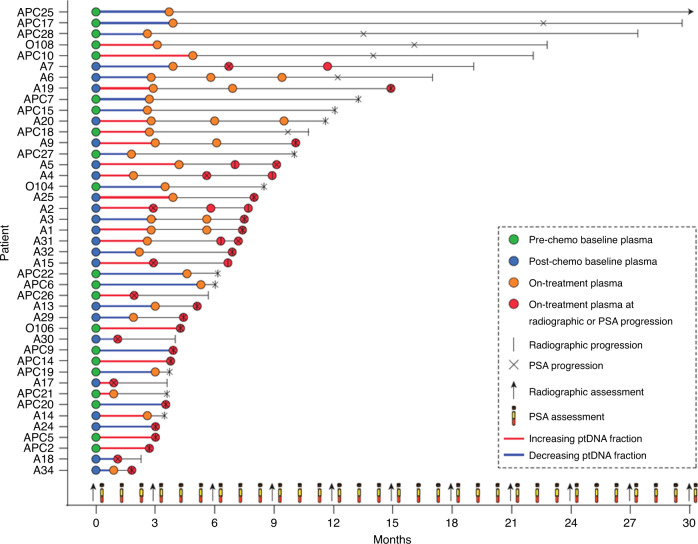
Overview of study samples and data points. Graph showing the time after start of treatment to plasma sample collection and progression by PSA or radiographic assessment for 21 chemotherapy-naive (Pre-chemo) and 22 post docetaxel (Post-chemo) patients.

Between January 2011 and June 2016, we collected 114 plasma samples from 43 mCRPC patients treated with abiraterone (21 chemotherapy naive and 22 post docetaxel) at Istituto Scientifico Romagnolo per lo Studio e la Cura dei Tumori (IRST) IRCCS, Meldola, Italy (Fig. 1). Overall, median age was 74 years (interquartile range (IQR) 70–78). Nine (20.9%) patients had visceral metastasis. Chemotherapy-naive patients had a significantly lower incidence of Gleason score ≥8 at diagnosis (*p* = 0.005) and higher levels of baseline haemoglobin (*p* = 0.0003) levels compared to post-docetaxel-treated group (Table 1). The first on-treatment plasma samples were taken at a median of 3 months after treatment (IQR = 2.6–3.7, Supplementary Fig. 1).

**Table 1:** Patient characteristics.

	Total (*n* = 43)	Chemotherapy naive (*n* = 21)	Post docetaxel (*n* = 22)	*p* Value
Age (years)	74	76	73	
Median (IQR)	(70–80)	(69–81)	(72–77)	0.532
Gleason score, *n* (%)				
<8	15 (38.5)	12 (60.0)	3 (15.8)	
≥8	24 (61.5)	8 (40.0)	16 (84.2)	0.005
Unknown/missing	4	1	3	
Bone mts, *n* (%)				
No	8 (18.6)	5 (23.8)	3 (13.6)	
Yes	35 (81.4)	16 (76.2)	19 (86.4)	
<6	14	3	11	
>6	21	13	8	0.514
Visceral mts, *n* (%)	9 (20.9)	2 (9.5)	7 (31.8)	0.076
Liver mts, *n* (%)	2 (4.6)	1 (4.7)	1 (4.5)	0.973
Lymph node mts, *n* (%)	20 (46.5)	11 (52.4)	9 (40.9)	0.456
Baseline PSA (ng/mL)	40.54	32.76	54.05	
Median (IQR)	(10.17–81.39)	(1.34–403.50)	(11.44–123.40)	0.174
Baseline LDH (U/L)	168	167	177	0.449
Median (IQR)	(151–201)	(145–198)	(157–208)	
Baseline ALP (IU/L)	98	96	103	0.449
Median (IQR)	(85–133)	(87–121)	(71–133)	
Baseline haemoglobin (g/dL)	13.9	14.0	13.1	0.0003
Median (IQR)	(13.2–14.2)	(14.0–14.6)	(12.2–13.9)	
Baseline albumin (g/dL)	4.1	4.1	3.9	0.220
Median (IQR)	(3.8–4.2)	(3.9–4.3)	(3.5–4.1)	
Baseline NLR (*n* (%))				
<3	23 (53.5)	13 (61.9)	10 (45.5)	
≥3	20 (46.5)	8 (38.1)	12 (54.5)	0.285

*ALP* alkaline phosphatase, *IQR* interquartile range, *LDH* lactate dehydrogenase, *mts* metastasis, *n* number, *NLR* neutrophil-to-lymphocyte ratio, *PSA* prostate-specific antigen.

### Early changes in ptDNA and radiographic and biochemical response

**Fig. 2:**
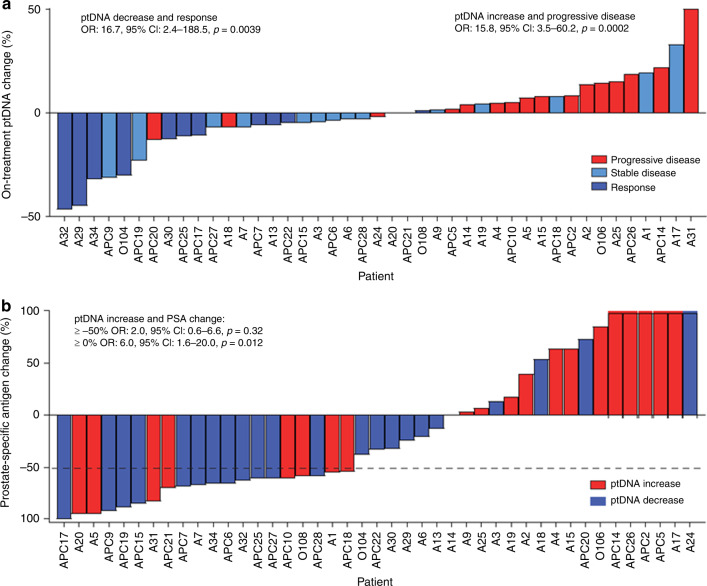
Early changes in ptDNA and radiographic and biochemical response. Waterfall plot of ptDNA change and **a** radiographic response and **b** PSA change in on-treatment vs. baseline samples. PSA change was capped at 100%; dashed line indicates PSA change of −50%. Odds ratio was calculated using Baptista–Pike and *p* value by Fisher’s exact test.

**Fig. 3:**
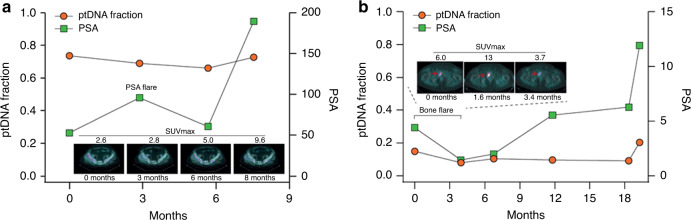
Association of ptDNA dynamics with treatment response. **a** ptDNA change and PSA flare in patient A3. **b** ptDNA change and bone pseudoprogression in patient A7. Patient A3 is characterised by an initial PSA elevation followed by a PSA decline and no radiographic progression on 18F-fluorocholine positron emission tomography/computed tomography (FCH-PET/CT) by using semiquantitative criteria based on the maximum standardised radiotracer uptake value (SUVmax). Patient A7 showed a bone flare on abiraterone characterised by an early increase in the intensity of bone lesions in the context of treatment response. In both these cases, ptDNA did not follow PSA and radiographic changes, but reduced in the first ~3 months from starting abiraterone.

We examined the correlation between ptDNA fraction and disease status at first restaging in patients who completed at least 3 months of abiraterone. Supplementary Fig. 2 shows an overview of changes between the baseline and on treatment ptDNA fractions. We observed that patients with an increase in ptDNA fraction had a significantly increased risk of having PD, based on 3-month early radiographic assessment (OR = 15.8, 95% CI 3.5–60.2, *p* = 0.0002, Fig. 2a). Conversely, patients with a decrease in ptDNA fraction had significantly higher chance of having a response (OR = 16.7, 95% CI 2.4–188.5, *p* = 0.0039, Fig. 2a). Moreover, patients with an increase in ptDNA fraction had an increased risk of having a PSA rise (OR = 6.0, 95% CI = 1.6–20, *p* = 0.012, Fig. 2b). We did not observe an association between on-treatment ptDNA fraction increase and the absence of a ≥50% PSA decline (OR = 2.0, 95% CI = 0.6–6.6, *p* = 0.32). Interestingly, we identified examples of cases with a PSA flare or pseudoprogression on imaging secondary to a bone flare, but without a rise in ptDNA fraction (Fig. 3). We performed a supplementary analysis to evaluate the association between baseline ptDNA and clinical outcome. Patients were dichotomised into two groups according to the ptDNA fraction cut-off of 0.18 assessed by receiver-operating characteristic curve analysis. Patients with a ptDNA fraction >0.18 had significantly shorter PFS and a trend towards shorter OS (*p* = 0.02 and *p* = 0.10, respectively, Supplementary Fig. 3).

### Impact of previous treatment on ptDNA fraction change

**Fig. 4:**
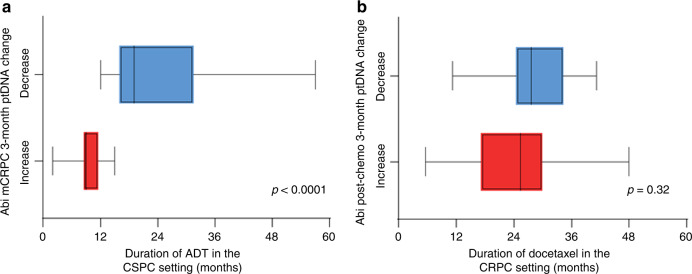
Previous response to different treatments according to ptDNA increase or decrease upon abiraterone treatment. **a** Previous response to ADT at hormone-sensitive prostate cancer (HSPC) setting in patients with ptDNA increase or decrease upon abiraterone treatment. **b** Previous response to docetaxel at castration-resistant prostate cancer (CRPC) setting in patients with ptDNA increase or decrease upon abiraterone treatment.

All patients underwent androgen deprivation treatment (ADT) with an LHRH agonist and antiandrogens before abiraterone. We found that the ptDNA change with abiraterone was significantly associated with previous duration of ADT (Fig. 4a). This was observed both for chemotherapy-naive and post-docetaxel patients (Supplementary Fig. 4). However, no association was seen with previous docetaxel response in the post-docetaxel patients (Fig. 4b).

## DISCUSSION

One key aim of treating CRPC is to prolong survival while maintaining quality of life and preventing needless toxic effects of ineffective treatments. Currently, tumour response and treatment benefit are determined using radiographic imaging and PSA assessment, but this may result in patient over-treatment and can be confounded by an apparent initial worsening that is in fact due to a flare secondary to bone remodelling at responding metastatic sites. Our study shows the potential of adding ptDNA assessment to routine monitoring of mCRPC patients.

Of note, a rise in ptDNA was strongly associated with radiological progression or any PSA rise, but not with PSA or radiological flares, indicating potential added value.^16,17^ Future prostate cancer studies utilising more frequent plasma sampling should enable the identification of any lead time benefit of ptDNA assessment for progression, as has been observed in other cancers.^18,19^

In our study, there was no association with ptDNA change and ≥50% PSA decline. Although an exploratory study, the association of ptDNA change on abiraterone with previous response to ADT at castration-sensitive prostate cancer (CSPC) provides an additional level of support to the relevance of early ptDNA assessment and suggests that patients with rapid progression on ADT are less likely to derive benefit from subsequent androgen signalling targeting even when separated by one line of treatment.

The main limitations of the study are the small cohort of patients, including both taxane-naive and post-docetaxel patients, the retrospective nature of the analysis and the limited number of multiple sequential plasma samples. Another general limitation for ptDNA studies is how to define progression. As we had a baseline and an on-treatment sample for all patients, we took a pragmatic approach and considered any increase of ptDNA fraction as progression. For future studies, we would envision a combination of a cut-off for what is considered a ptDNA change as well as having two subsequent samples showing an increased ptDNA fraction to confirm ptDNA rise. Despite these limitations, the present study represents a first step towards achieving the introduction of longitudinal ptDNA fraction assessment with standard tests utilised in clinical practice. This work underlines the potential of minimally invasive liquid biopsies to better inform future decision making that have suggested circulating tumour cell detection. Serial ptDNA analysis of prostate cancer patients is now being incorporated into larger prospective clinical studies.

## CONCLUSION

In this report, we looked at the utility of ptDNA monitoring during abiraterone treatment. We found that ptDNA assessment associated with radiological progression or response and any decline in PSA, but also provided independent information. Future studies could support the integration of ptDNA into composite biomarker tests for early assessment of treatment benefit.

## Supplementary information

Supplemental Material

## Data Availability

All data generated or analysed during this study are included in this published article and its Supplementary information data
