# Supplementary material for: Plasma tumour DNA as an early indicator of treatment response in metastatic castration-resistant prostate cancer
Source: Br J Cancer. 2020 Jul 16;123(6):982–7. doi: 10.1038/s41416-020-0969-5 (PMC7492429; doi:10.1038/s41416-020-0969-5)
Supplement: Supplementary file 1 — Supplemental Material [file 41416_2020_969_MOESM1_ESM.docx]

**Supplementary Online Material**

**Supplementary Figure 1.** Time to first on-treatment plasma samples.….………..…………… ……….….….2

**Supplementary Figure 2**. Overview of changes between the baseline and on treatment plasma tumour (ptDNA) DNA fractions……………………………………………….………………………………………………..3

**Supplementary Figure 3.** Association of plasma tumour DNA (ptDNA) fraction before abiraterone with treatment outcome………………………………………………………………………..………………..………….4

**Supplementary Figure 4.** Previous response to ADT at hormone sensitive prostate cancer………………..5


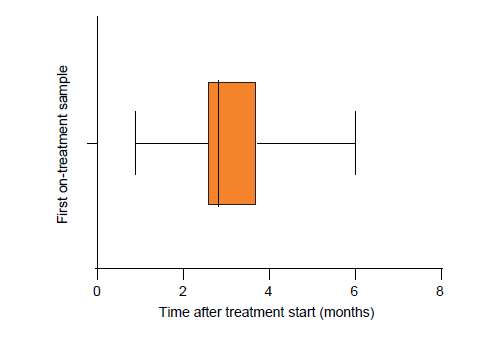


**Supplementary Figure 1.** Time to first on-treatment plasma samples.


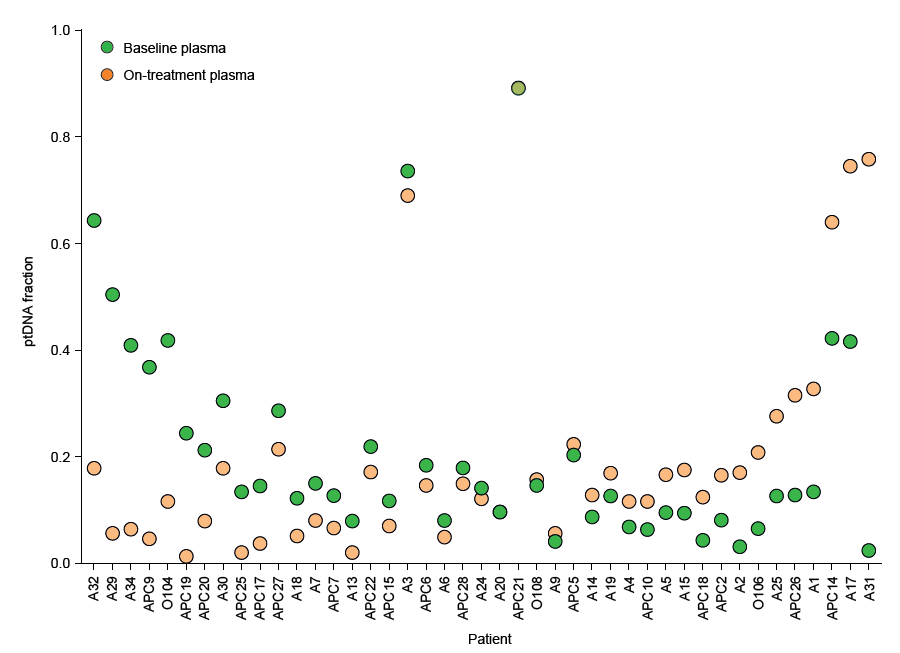


**Supplementary Figure 2**. Overview of changes between the baseline and on treatment ptDNA fractions.


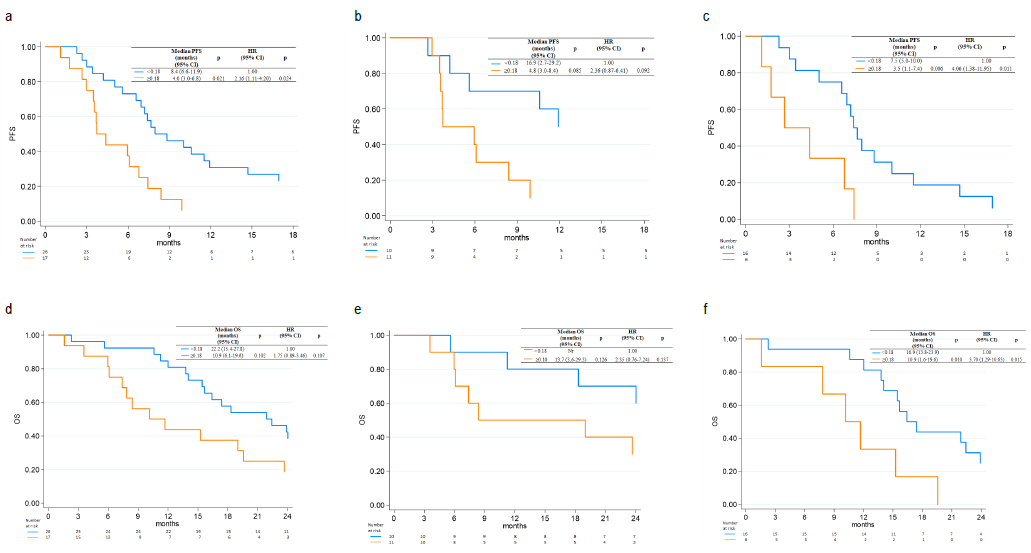


**Supplementary Figure 3.** Association of plasma tumour DNA (ptDNA) fraction before abiraterone with treatment outcome. Progression-free survival (PFS) in overall (a), chemotherapy-naive (b) and post-chemotherapy (c) patients according to median baseline ptDNA fraction. Overall survival (OS) in overall (d), chemotherapy-naive (e) and post-chemotherapy (f) patients according to median value of baseline ptDNA.


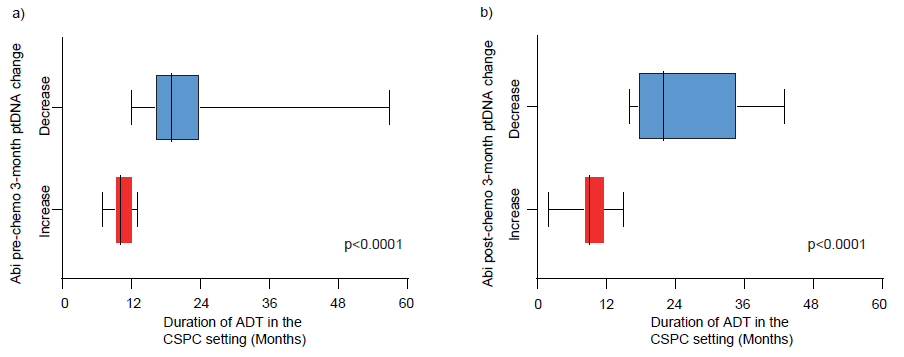


**Supplementary Figure 4.** (a) Previous response to ADT at hormone-sensitive prostate cancer (HSPC) in pre-chemotherapy and (b) in post-chemotherapy patients with ptDNA increase or decrease upon abiraterone treatment.
